# Retrospective observation of drug susceptibility of *Candida* strains in the years 1999, 2004, and 2015

**DOI:** 10.7717/peerj.3038

**Published:** 2017-02-23

**Authors:** Cecylia Łukaszuk, Elżbieta Krajewska-Kułak, Wojciech Kułak

**Affiliations:** 1 Department of Integrated Medical Care, Medical University of Bialystok, Białystok, Poland; 2 Department of Pediatric Rehabilitation, Medical Univeresity of Białystok, Białystok, Poland

**Keywords:** *Candida*, 5 years, Retrospective study, Drug susceptibility, Resistance

## Abstract

**Introduction:**

There is much literature devoted to the problem of drug resistance and decreased susceptibility of fungi to commonly used antifungals.

**Aim:**

To analyze drug susceptibility of *Candida albicans* and *non-Candida albicans* strains isolated from the hands of people without any symptoms of disease over a 16-year period.

**Materials and Methods:**

The study included a total of 1,274 *Candida-type strains* isolated from the hands of people without any symptoms of disease*,* including: in 1999, 432 strains; in 2004, 368; and in 2015, 454 strains. Biological monitoring of hand surface contamination was performed using the Count-Tact^TM^ applicator with Count-Tact plates (bioMerieux). Drug susceptibility was evaluated using FUNGITEST^®^.

**Results:**

In 1999, the most strains showed resistance to fluconazole (53.2%), in 2004 to itraconazole (52.9%), and in 2015 to fluconazole (85.8%). Resistance to more than one drug was 35.8% in 1999, 64.7% in 2004, and 92% in 2015. Mean resistance to azole antifungals significantly increased from 98 ± 39.7 strains in 1999 to 118.3 ± 29.6 in 2015 (*p* < 0.001). In 1999, the most strains showed resistance to fluconazole (50.6%), in 2004 to itraconazole (52.9%), and in 2015 to fluconazole (44.9%). Resistance to more than one drug was 52.9% in 1999, 64.3% in 2004, and 88.1% in 2015. Mean resistance to azole antifungals significantly increased from a mean of 76 ± 9.7 strains in 1999, to 95.3 ± 24.2 in 2004, and to 97.3 ± 16.6 in 2015 (*p* < 0.001).

**Conclusions:**

We showed increased *C. albicans* and *non-Candida albicans* strain resistance to commonly used antifungal chemotherapeutics, mainly imidazole. We found a clear rise in susceptibility of *C. albicans* and *non-Candida albicans* strains to several studied antifungals.

## Introduction

A phenomenon constantly emphasized in the literature is the emergence of yeast-like fungi strains resistant to individual drugs or whole groups of antifungals (Pfaller et al., 1998; Tsai et al., 2006; Bailly et al., 2016; Ben-Ami et al., 2016; Zaidi et al., 2016; Sanglard, 2016). There are two types of resistance in fungi: primary and secondary (induced) (Sanguinetti, Posteraro & Lass-Flörl, 2015). Primary resistance pertains to cells with high Minimal Inhibitory Concentration (MIC) values which had no prior contact with a given antimycotic drug. Secondary resistance is associated with strains that were originally susceptible and acquired resistance through induction (or selection of naturally resistant mutants). Cases of primary (about 10–20% strains) and secondary resistance have been widely described in relation to 5-fluorocytosine. *Candida glabrata* and *Candida krusei*, for example, show primary resistance to fluconazole (Richardson & Warnock, 1995). The most common strains resistant to amphotericin B are *Candida parapsilosis*, *Candida lusitaniae*, *Candida quillermondii*, *Candida tropicalis*, and *Candida krusei*. According to some authors, increased drug resistance could be associated with improper treatment (Vanden Bossche et al., 1998).

*Candida* strain antifungal resistance is the result of antimycotic drug use for treating fungal infections, especially during preventive and empiric therapy, causing resistant strain selection (Tsimbalari et al., 2015). Most likely to occur during therapy, yeast-like fungi, which are etiologic factors of infections, may be replaced by other species derived from hospital flora, resistant to the drugs used. *Candida* spp. clinical isolates commonly exhibit a high inherent tolerance level to azole antimycotics. Azole resistance can be acquired through an increased expression of genes encoding ABC transporters (Cdr1, Pdh1, Snq2) or changes in their transcriptional regulatory system (Pdr1, Gal11) (Thakur et al., 2008). Mitochondrial dysfunction and serum utilization via the putative sterol transporter Aus1 also impact the ability of *Candida* spp. to tolerate high azole levels (Brun et al., 2004; Nagi et al., 2013). In addition, calcineurin signaling has been implicated in azole tolerance in *Candida*. Therefore, research on the acquisition of genes determining resistance is becoming more common (Macura & Skóra, 2012; Kabir & Ahmad, 2013). There is also a need to observe and monitor changes in antifungal activity.

While antibiotic-resistant bacterial infections are a widely-recognized public health threat, less is known about the effects of antifungal resistance and the burdens caused by drug-resistant fungal infections. These invasive infections cause considerable morbidity and mortality and are common problems in healthcare settings (Magill et al., 2014; Mohamadi et al., 2015). The fungus *Candida* is the most common cause of healthcare-associated bloodstream infections in the United States (Magill et al., 2014). Each case of *Candida* infection of the bloodstream is estimated to result in an additional three to 13 days of hospitalization and thus increases healthcare costs significantly (Morgan et al., 2005). Even with current antifungal therapy, mortality associated with candidiasis can be as high as 50% in adults and up to 30% in children (Moran et al., 2009).

It is known that the hands of healthcare workers are responsible for 20–40% of nosocomial infections (Rożkiewicz, 2011). Moreover, the hands of medical students can be a route of transmission of microorganisms.

Healthy people and healthcare workers can also carry *Candida* on their hands (Yildirim et al., 2007). There have been outbreaks of candidemia linked to healthcare workers’ hands (Clark et al., 2004); therefore, hand hygiene in healthy people and healthcare settings is critical for preventing the spread of infections.

Thus, monitoring people’s hands for the presence of *Candida* spp. is important. The aim of this study was to analyze, over the course of sixteen years, drug susceptibility and resistance of yeast-like fungi strains isolated from the hands of people with no disease symptoms.

## Materials

Participation in the study was voluntary. A total of 667 students of the Medical University of Białystok, Poland took part in the study. Respondents’ ages ranged from 19 to 25 (22 ± 2.3). The dominant hands of the participants without clinical symptoms were sampled in the morning.

The study included a total of 1,274 *Candida-*type strains isolated from the hands of people without any symptoms of disease, including the following. In 1999, 432 strains from 229 students; in 2004, 368 from 198 students; and in 2015, 454 strains from 240 students were isolated. To facilitate the analysis of statistical dependence, the studied strains were divided into two groups: I—*C. albicans* and II—*non-Candida albicans*. Group I had 630 *C. albicans* strains, including 282 strains from 1999, 172 from 2004, and 176 from 2015. Group II had 644 *non-Candida albicans* strains, including 170 from 1999, 196 from 2004, and 278 from 2015.

### Identification yeast-like fungi

Biological monitoring of hand surface contamination was performed using the Count-Tact™ applicator with Count-Tact plates (bioMerieux, Marcy l’Etoile, France) containing a medium complying with the Draft European Standard CEN/TC 243/WG2 requirements. CandiSelect (Bio-Rad, Hemel Hempstead, UK) was used to identify yeast-like fungi. It is a selective chromogenic medium designed for the isolation of yeasts, the direct identification of *C. albicans* and the presumptive identification of *C. tropicalis, C. glabrata* and *C. krusei*.

After sampling, Count-Tact plates were incubated at 37C up to 48 h. Next, the fungal culture was inoculated into CandiSelect. The plates were incubated for 72 h to allow for identification. The color and intensity of colonies was assessed every 24 h. *C. albicans* produced pink to purple colored colonies; *C. glabrata* produced turquoise colonies that were shiny and flat with a regular outline; *C. tropicalis* produced intense turquoise colored colonies that were spherical with a regular outline; C. *krusei* produced large turquoise colonies with a dry appearance and an irregular outline.

### Drug concentration

Drug susceptibility was assessed using FUNGITEST^®^ (Sanofi Diagnostics Pasteur, Paris, France in the years, 1999, 2004 since 2015; Bio-Rad, Marnes-la-Coquette, France) (Witthuhn et al., 1999) to analyze yeast-like fungal growth in the presence of two concentrations of six drugs: 5-fluorocytosine, amphotericin B, miconazole, ketoconazole, itraconazole and fluconazole, in modified RPMI 1640 medium, in the presence of a redox indicator. Growth assessment is based on the reduction of the colored indicator which turns the medium from blu**e** to pink. When growth is inhibited by the antifungal agent, the medium remains blue. This test, presented in the form of a 16-well microplate, consists of two growth control wells; 12 wells containing the dehydrated antifungal agents (six antifungal agents at two different concentrations); 5-fluorocytosine (2–32 µg/ml), amphotericin B (2–8 µg/ml), miconazole (0.5–8 µg/ml) kétoconazole (0.5–4 µg/ml), itraconazole (0.5–4 µg/ml), fluconazole (8–64 µg/ml); two negative control wells. The breakpoints have been chosen following the study of the distribution of the antifungal agents M.I.C’s obtained with prototype microplates used with the same procedure as Fungitest. Results were interpreted in accordance with the manufacturer’s instructions, always by the same person, and always with reference to the color of two wells containing the same drug: a blue color in both wells indicated an *in vitro* susceptible strain; a pink color at lower concentrations and a blue color at higher concentrations indicated an *in vitro* strain with low susceptibility; and a pink color in both wells indicated an *in vitro* resistant strain.

The study has been accepted by ethic committee of the Medical University of Białystok, Poland, approval numbers: R-I-003/64/99; R-I-003/222/2004, and RI-002/489/2010.

## Statistical Analysis

Statistical analysis of the results was done using the chi^2^ test and the Kruskal–Wallis test on Statistica 10.0 software.

## Results

Generally, *C. albicans* strains were most susceptible to 5-fluorocytosine (67.8%) and least susceptible to itraconazole (14.1%). The *C. albicans* strains were most resistant to fluconazole (52.9%) and least resistant to amphotericin B (6.2%). Generally, the most *non-Candida albicans* strains were most susceptible to 5-fluorocytosine (69.7%), and least susceptible to ketoconazole (27.6%). The *non-Candida albicans* were most resistant to ketoconazole (50.5%) and least susceptible to amphotericin B (5.9%). Details are presented in Table 1.

**Table 1:** Drug susceptibility of *Candida* strains.

Results	DRUGS					
	*5-fluorocytosine*	*Amphotericin B*	*Miconazole*	*Ketoconazole*	*Itraconazole*	*Fluconazole*
Susceptibility						
*Candida albicans* *N* = 630	**427 (67.8%)**	399 (63.3%)	192 (30.5%)	165 (26.2%)	89 (14.1%)	145 (23%)
*Non-Candida albicans* *N* = 644	**449 (69.7%)**	409 (63.5%)	206 (32.98%)	178 (27.6%)	211 (32.8%)	192 (29.8%)
*P* value^*^	0.781	<0.01	0.716	0.706	<0.001	<0.05
Intermediate						
*Candida albicans* *N* = 630	144 (23.9%)	192 (30.5%)	**227 (36%)**	**227 (36%)**	183 (29%)	112 (17.8%)
*Non-Candida albicans* *N* = 644	144 (22.4%)	197 (30.6%)	**241 (37.4%)**	141 (21.9%)	146 (22.7%)	**147 (22.8%)**
*P* value^*^	0.917	0.979	0.767	<0.001	<0.05	0.656
Resistance						
*Candida albicans* *N* = 630	59 (9.4%)	39 (6.2%)	211 (33.5%)	238 (37.8%)	358 (56.8%)	**373 (59.2%)**
*Non-Candida albicans* *N* = 644	51 (7.9%)	38 (5.9%)	**197 (30.6%)**	**325 (50.5%)**	287 (44.6%)	**305 (47.3%)**
*P* value^*^	0.457	0.932	0.428	<0.01	<0.05	<0.001

**Notes.**

* Test Chi^2^ test.

In 1999, the *C. albicans* were most resistant to fluconazole (53.2%), and the least resistant to amphotericin B (1.1%). In 2004, we found the *C. albicans* were most resistant to itraconazole (52.9%), and least resistant to amphotericin B (3.5%). In 2015, the most *C. albicans* showed resistance to fluconazole (85.8%), and least resistance to amphotericin B (17%). Results are presented in Table 2. We observed statistically significant differences between the analyzed years pertaining to the studied *C. albicans* strains:

• in susceptibility to amphotericin B (*p* = 0.011), ketoconazole (*p* = 0.016), itraconazole (*p* < 0.001), and fluconazole (*p* < 0.001)
• in decreased susceptibility to all drugs
• in resistance to all drugs.

Results are presented in Table 2.

**Table 2:** Drug susceptibility of studied *Candida albicans* strains by year.

Results		DRUGS					
		*5- fluorocytosine*	*Amphotericin B*	*Miconazole*	*Ketoconazole*	*Itraconazole*	*Fluconazole*
Susceptibility/year	1999 *N* = 282	**231 (81.9%)**	229 (81.2%)	112 (39.7%)	114 (40.4%)	68 (24.1%)	96 (34%)
	2004 *N* = 172	**99 (57.6%)**	94 (54.6%)	12 (16.7%)	8 (4.6%)	4 (2.3%)	29 (16.9%)
	2015 *N* = 176	**97 (55.1%)**	76 (43.2%)	68 (38.6%)	43 (24.4%)	17 (11.8%)	20 (11.4%)
	*P* value^*^	0.08	0.011	0.603	0.016	<0.001	<0.001
Intermediate/year	1999 *N* = 282	46 (16.3%)	50 (17.7%)	109 (38.7%)	**110 (39%)**	91 (32.3%)	36 (12.8%)
	2004 *N* = 172	53 (30.8%)	72 (41.9%)	**94 (54.6%)**	78 (45.3%)	77 (44.8%)	71 (41.3%)
	2015 *N* = 176	45 (25.6%)	**70 (39.8%)**	24 (13.6%)	39 (22.2%)	15 (8.5%)	5 (2.8%)
	*P* value^*^	<0.01	<0.001	<0.001	<0.01	<0.001	<0.001
Resistance/year	1999 *N* = 282	5 (1.8%)	3 (1.1%)	61 (21.6%)	58 (20.6%)	123 (43.6%)	**150 (53.2%)**
	2004 *N* = 172	20 (11.6%)	6 (3.5%)	66 (38.4%)	86 (50%)	**91 (52.9%)**	72 (41.9%)
	2015 *N* = 176	34 (19.3%)	30 (17%)	84 (47.7%)	94 (53.4%)	144 (81.8%)	**151 (85.8%)**
	*P* value^*^	<0.001	<0.001	<0.001	<0.001	<0.001	<0.01

**Notes.**

* Kruskal–Wallis test.

In 1999, the *non-Candida albicans* was most resistant to ketoconazole (72.9%), and least resistant to amphotericin B (10.6%). In 2004, we detected that *non-Candida albicans* were most resistant to itraconazole (58.2%), and least resistant to 5-fluorocytosine (5.1%). In 2015, the *non-Candida albicans* showed most resistance to fluconazole (44.9%), and the least resistant to amphotericin B (1.4%). Results are presented in Table 3.

**Table 3:** Drug susceptibility of studied *non-Candida albicans* strains by year.

Results *non Candida albicans*		**DRUGS**					
		*5-fluorocytosine*	*Amphotericin B*	*Miconazole*	*Ketoconazole*	*Itraconazole*	*Fluconazole*
Susceptibility/year	1999 *N* = 170	**120 (70.6%)**	110 (65.3%)	44 (25.9%)	22 (12.9%)	52 (30.6%)	56 (32.9%)
	2004 *N* = 196	**148 (75.5%)**	104 (53.01%)	46 (23.5%)	52 (25.6%)	50 (25.5%)	64 (32.7%)
	2015 *N* = 278	**181 (65.1%)**	195 (70.1%)	116 (56.1%)	104 (37.4%)	109 (39.2%)	72 (25.9%)
	*P* value^*^	0.650	0.654	<0.05	<0.001	0.580	0.278
Intermediate/year	1999 *N* = 170	16 (9.4%)	42 (24.7%)	**66** (38.8%)	24 (14.1%)	38 (22.4%)	36 (21.2%)
	2004 *N* = 196	38 (19.4%)	76 (38.7%)	**96 (49%)**	33 (16.8%)	32 (16.3%)	30 (15.3%)
	2015 *N* = 278	**90 (32.4%)**	79 (28.4%)	79 (28.6%)	84 (30.2%)	76 (27.3%)	81 (29.1%)
	*P* value^*^	<0.001	0.584	0.128	<0.01	0.422	0.181
Resistance/year	1999 *N* = 170	34 (20%)	18 (10.6%)	60 (35.3%)	**124 (72.9%)**	80 (47.1%)	78 (45.9%)
	2004 *N* = 196	10 (5.1%)	16 (8.2%)	54 (56.3%)	111 (56.6%)	**114 (58.2%)**	102 (52%)
	2015 *N* = 278	7 (2.5%)	4 (1.4%)	**83 (29.6%)**	90 (32.4%)	93 (33.5%)	**125 (44.9%)**
	*P* value^*^	<0.001	<0.001	0.374	<0.05	0.07	0.946

**Notes.**

* Kruskal–Wallis test.

We observed statistically significant differences between the analyzed years pertaining to *non-Candida albicans* strains:

• in susceptibility to miconazole (*p* < 0.05) and ketoconazole (*p* < 0.001)
• in decreased susceptibility to 5-fluorocytosine (*p* < 0.001) and ketoconazole (*p* < 001)
• in resistance to 5-fluorocytosine (*p* < 0.001), amphotericin B (*p* < 0.001), and ketoconazole (*p* < 0.05).

Results are presented in Table 3.

Generally, 60.2% of *C. albicans* strains showed resistance to one or more of the studied antifungals, and most often to three drugs (24%). Resistance to more than one drug was 35.8% in 1999, 64.7% in 2004, and 92% in 2015. Results are presented in Table 4.

**Table 4:** Drug resistance of *Candida albicans* strains to one or more antifungal.

Drugs	Year			Total *N* = 630
	1999 *N* = 282	2004 *N* = 172	2015 *N* = 176	
One or more	101 (35.8%)	116 (67.4%)	**162 (92%)**	379 (60.2%)
*P* value^*^	<0.001			
1	0	2 (1.2%)	**81 (46%)**	83 (13.2%)
*P* value^*^	<0.001			
2	**18 (6.4%)**	4 (2.3%)	14 (8%)	36 (5.7%)
*P* value^*^	0.550			
3	**61 (21.6%)**	41 (23.8%)	49 (27.8%)	**151 (24%)**
*P* value^*^	0.826			
4	22 (7.8%)	**61 (35.5%)**	12 (6.8%)	95 (15.1%)
*P* value^*^	0.857			
5	0	**8 (4.7%)**	6 (3.4%)	14 (2.2%)
*P* value^*^	<0.01			

**Notes.**

* Kruskal–Wallis test.

Generally, 71.8% of *non-Candida albicans* strains showed resistance to one or more of the studied antifungals, and most often to one (26%) or two drugs (25.7%). Resistance to more than one drug was 52.9% in 1999, 64.3% in 2004, and 88.1% in 2015. Results are presented in Table 5.

**Table 5:** Resistance of *non-Candida albicans* strains to one or more antifungal drug.

Drugs	Year			Total *N* = 644
	1999 *N* = 170	2004 *N* = 196	2015 *N* = 278	
One or more	90 (52.9%)	126 (64.3%)	**245 (88.1%)**	461 (71.8%)
*P* value^*^	<0.01			
1	**45 (64.3%)**	21 (10.7%)	101 (36.3%)	**167 (26%)**
*P* value^*^	0.112			
2	6 (3.5%)	34 (17.3%)	**125 (44.9%)**	165 (25.7%)
*P* value^*^	<0.001			
3	14 (8.2%)	**37 (18.9%)**	7 (2.5%)	58 (9%)
*P* value^*^	¡ 0.05			
4	21 (12.4%)	**26 (13.3%)**	2 (0.7%)	49 (7.6%)
*P* value^*^	<0.001			
5	4 (2.4%)	8 (4.1%)	10 (3.6%)	22 (3.4%)
*P* value^*^	<0.05			

**Notes.**

* Kruskal–Wallis test.

We found significant differences between the analyzed years and resistance of the studied *C. albicans* and *non-Candida albicans* strains to one or more antifungal drugs (*p* < 0001). Generally, 295 ± 71.3 *C. albicans* strains were resistant to all studied antifungals. The mean resistance of *C. albicans* strains to antifungals significantly increased: 98 ± 39.7 in 1999, 118.3 ± 29.6 in 2015 (*p* < 0.001). Details are shown in File S1.

Generally, 259 ± 50.2 of *non-Candida albicans* strains were resistant to the studied antifungals. The mean resistance of *non-Candida albicans* to the studied antifungals significantly increased: 85.5 ± 23.6 of strains in 1999, 95.3 ± 24.2 in 2004, and 97.3 ± 16.6 in 2015 (<0.001). Details are shown in File S1.

We observed an increase of resistance to: Miconazole: 35.3% of resistant strains in 1999, 56.3% in 2004, and decreased to 29.6% in 2015 (*p* = 0.399), Ketoconazole 72.9% of resistant strains in 1999, 56.6% in 2004, and decreased to 32.4% in 2015 (*p* < 0.05), Itraconazole 47.1% of resistant strains in 1999, 58.2% in 2004, and decreased to 33.5% in 2015 (*p* = 0.07) and Fluconazole 45.9% of resistant strains in 2004, 52% in 2015, and decreased to 44.9% in 2015 (*p* = 0.999). We found significant (*p* < 0.001) differences in resistance to the studied antifungals between *C. albicans* and *non-Candida albicans* strains.

## Discussion

In the present study, we found increased *C. albicans* and *non-Candida albicans* strain resistance to commonly used antifungal chemotherapeutics, mainly imidazole. We also demonstrated an increase in the susceptibility of *C. albicans* and *non-Candida albicans* strains to several studied antifungals. Our findings are in accordance with previous studies (Batura-Gabryel, Wieczorek & Młynarczyk, 1997; Kubicka-Musiałet al., 2011; Macura & Skóra, 2012; Araj, Asmar & Avedissian, 2015; Bailly et al., 2016).

Kubicka-Musiałet al. (2011) assessed the susceptibility to antifungals of *Candida* strains isolated from patients with burning symptoms of the oral mucosa. Most *C. albicans* strains were susceptible to amphotericin B (94%), nystatin (91.6%), and flucytosine (94%). The authors found low susceptibility of the investigated strains to econazole, ketoconazole, miconazole, and fluconazole. According to various authors, the percentage of strains resistant to amphotericin B oscillates from 29 to 100 (Batura-Gabryel, Wieczorek & Młynarczyk, 1997; Swoboda-Kopeć et al., 1998; Dworecka-Kaszak, 2010).

In our study, *C. albicans* strain resistance to amphotericin B significantly increased. This strain resistance pertained to 6.2% strains; however, over the course of 16 years, it increased from 1.1% in 1999, to 3.5% in 2004, followed by 17% in 2015. In the case of *non-Candida albicans*, resistance to this drug significantly decreased. This resistance pertained to 5.9% strains; over the course of 16 years, it decreased from 10.6% in 1999, through 8.2% in 2004, to 1.4% in 2015.

According to Vanden Bossche et al. (1998), the resistance of *Candida* spp. strains to 5-fluorocytosine develops during the course of monotherapy using this drug. The authors observed a simultaneous increase in resistance to 5-fluorocytosine and itraconazole among strains. In the study by Cybulski et al. (2003), resistance to 5-fluorocytosine was determined in 8.3% *C. glabrata* strain isolates from the hospital patients.

Oberoi et al. (2012) emphasized that the incidence of candidemia caused by non-albicans infections has increased significantly and is correlated with the increased use of fluconazole. The authors observed a cross-resistance or reduced susceptibility to fluconazole and voriconazole in 11.3% of isolates.

Macura & Skóra (2012) assessed the susceptibility of fungi isolated from the vagina to six antifungals (5-fluorocytosine, amphotericin B, miconazole, ketoconazole, itraconazole, and fluconazole) using the Fungitest. The authors found that *C. krusei* had the highest resistance to antifungal drugs, including fluconazole. Of 23 *C. krusei* strains isolated from patients with the suspicion of vaginal mycosis, 4.3% showed susceptibility and 87% moderate susceptibility to 5-fluorocytosine; 8.7% susceptibility and 69.6% moderate susceptibility to fluconazole; while 17.4% showed resistance to amphotericin B.

The observations by Witthuhn et al. (1999) on the high resistance to fluconazole are interesting. The authors found 77% of strains were susceptible to fluconazole and 84% to itraconazole in HIV-infected patients.

In the present study, we found a rise in *C. albicans* strains resistant to fluconazole from 53.2% in 1999, through 41.9% in 2004, to 67.1% in 2015; and to itraconazole from 43.6% in 1999, through 52.9% in 2004, to 81.8% in 2015. In the case of *non-Candia albicans* strains, resistance to fluconazole pertained to 45.9% strains in 1999, 52% in 2004, and 44.9% in 2015.

In their analysis of susceptibility of *C. albicans* isolated in the years 1984–1993 and 1984–1997, Boschman et al. (1998) found that, up until 1993, all isolates showed susceptibility to fluconazole, ketoconazole, and miconazole. Since 1994, new drug resistant strains have gradually emerged.

We have also observed a significant rise in the percentage of resistance to all imidazole agents. In the case of *C. albicans* strains, an average of 295 ± 71.3 strains showed resistance to imidazoles, including mean 98 ± 39.7 in 1999, 78.8 ± 10.1 in 2004, and 118.3 ± 29.6 in 2015. In the case of *non-Candida albicans* strains, an average of 249 ± 39.6 strains showed resistance to imidazoles, including mean 76 ± 9.7 in 1999, 95.3 ± 24.2 in 2004, and 97.3 ± 16.6 in 2015.

It is alarming that 60.2% *C. albicans* and 78.1% *non-Candida albicans* strains showed resistance to one or more of the studied antifungals. *C. albicans* strains were most often resistant to three drugs, and resistance to more than one drug increased from 35.8% in 1999 to 92% in 2015. *Non-Candida albicans* strains were most often resistant to one drug (26%), and resistance to more than one drug rose from 52.9% in 1999 to 88.1% in 2015.

Araj, Asmar & Avedissian (2015) conducted a retrospective *in vitro* study on antifungal drug resistance of 186 *non-Candida albicans* and 61 *C. albicans* strains during three time periods: 2005–2007, 2009–2011, 2012–2014. The authors found a high resistance (35%–79%) to itraconazole. *C. albicans* strains exhibited a high susceptibility to fluconazole and voriconazole.

In an earlier study (Łukaszuk, Niczyporuk & Krawczuk-Rybak, 2000), we conducted a five-year observation of drug susceptibility of yeast-like fungi isolated from oral cavity ontogenesis in patients with cancer and candidiasis symptoms. The study included 326 fungal strains isolated from the material collected from oral cavity ontogenesis in cancer patients, who in 1995 had symptoms of active candidiasis and in whom 104 strains were found in 1999. The authors found a general rise in drug resistance of *C. albicans* from 15.1% in 1994 to 27.7% in 1999, and a decline in drug resistance of *C. species* from 26.2% in 1994 to 12.2% in 1999. In 1999, the development of resistance of *C. albicans* to 5-fluorocytosine was an interesting phenomenon. *C. albicans* strains showed a two-fold increase in resistance to two or more antimycotics over a five year period, as opposed to *C. species* isolates, which showed a nearly two-fold decrease in their resistance to two or more drugs.

In the present study, we demonstrated statistically significant differences between the studied years in *C. albicans* strain susceptibility to amphotericin B, itraconazole, and fluconazole as well as reduced susceptibility and resistance to all drugs; and in the case of *non-C. albicans* strains to itraconazole and fluconazole.

## Conclusions

1. We showed increased *Candida albicans* and *non-Candida albicans* strain resistance to commonly used antifungal chemotherapeutics, mainly imidazole.
2. We found a clear rise in susceptibility of *Candida albicans* and *non-Candida albicans* strains to several studied antifungals.

##  Supplemental Information

10.7717/peerj.3038/supp-1 Data S1 Data showing drug susceptibility and resistance of *Candida albicans* and *non-Candida albicans* strains isolated from the hands of people without any symptoms in the years 1999, 2004 since 2015 Drug susceptibility was assessed using FUNGITEST^®^ to analyze yeast-like fungal growth in the presence of six drugs: 5-fluorocytosine, amphotericin B, miconazole, ketoconazole, itraconazole and fluconazole.

10.7717/peerj.3038/supp-2 Data S2 Raw data Raw data of *Candida* and *non-Candida* resistance, susceptibility to antifungals in excel file.

10.7717/peerj.3038/supp-3 File S1 Resistance and susceptibility of *Candida albicans* and *non-Candida albicans* strains to antifungal drugs in the years 1999, 2004, and 2015

## Additional Information and Declarations

### Data Availability

The following information was supplied regarding data availability: The raw data has been supplied as a Supplementary File.

## References

[ref-1] Araj GF, Asmar RG, Avedissian AZ (2015). Candida profiles and antifungal resistance evolution over a decade in Lebanon. The Journal of Infection in Developing Countries.

[ref-2] Bailly S, Maubon D, Fournier P, Pelloux H, Schwebel C, Chapuis C, Foroni L, Cornet M, Timsit JF (2016). Impact of antifungal prescription on relative distribution and susceptibility of *Candida* spp.—trends over 10 years. Journal of Infection.

[ref-3] Batura-Gabryel H, Wieczorek U, Młynarczyk W (1997). Wrażliwość na leki przeciwgrzybicze *in vitro* szczepów Candida wyizolowanych od chorych na różne choroby układu oddechowego. Pneumonologia i Alergologia Polska.

[ref-4] Ben-Ami R, Zimmerman O, Finn T, Amit S, Novikov A, Wertheimer N, Lurie-Weinberger M, Berman J (2016). Heteroresistance to fluconazole is a continuously distributed phenotype among candida glabrata clinical strains associated with *in vivo* persistence. MBio.

[ref-5] Boschman CR, Bodnar UR, Tornatore MA, Obias AA, Noskin GA, Englund K, Postelnick MA, Suriano T, Peterson LR (1998). Thirteen-year evolution of azole resistance in yeast isolates and prevalence of resistant strains carried by cancer patients at a large medical center. Antimicrobial Agents Chemotherapy.

[ref-6] Brun S, Berges T, Poupard P, Vauzelle-Moreau C, Renier G, Chabasse D, Bouchara JP (2004). Mechanisms of azole resistance in petite mutants of *Candida glabrata*. Antimicrobial Agents and Chemotherapy.

[ref-7] Clark TA, Slavinski SA, Morgan J, Lott T, Arthington-Skaggs BA, Brandt ME, Webb RM, Currier M, Flowers RH, Fridkin SK, Hajjeh RA (2004). Epidemiologic and molecular characterization of an outbreak of *Candida parapsilosis* bloodstream infections in a community hospital. Journal of Clinical Microbiology.

[ref-8] Cybulski Z, Krzemińska-Jaśkowiak E, Grabiec A, Talaga Z (2003). Porównanie lekowrażliwości szczepów *C. glabrata*, *C. tropicalis*. Współczesna Onkologia.

[ref-9] Dworecka-Kaszak B (2010). Genetyczne podstawy oporności wielolekowej u grzybów. Mikologia Lekarska.

[ref-10] Kabir MA, Ahmad Z (2013). Candida infections and their prevention. ISRN Preventive Medicine.

[ref-11] Kubicka-Musiał M, Musiał ST, Wierucka-Młynarczyk B, Hüpsch-Marzec H (2011). Częstość występowania infekcji grzybiczej oraz ocena lekowrażliwości grzybów z rodzaju Candida u pacjentów z objawami pieczenia błony śluzowej jamy ustnej. Dental and Medical Problems.

[ref-12] Łukaszuk C, Krajewska-Kułak E, Niczyporuk W, Krawczuk-Rybak M (2000). Pięcioletnia obserwacja lekowrażliwości szczepów grzybów drożdżopodobnych u pacjentów onkologicznych. Mikologia Lekarska.

[ref-13] Macura AB, Skóra M (2012). Grzyby izolowane z pochwy i ich wrażliwość na leki przeciwgrzybicze. Ginekologia Polska.

[ref-14] Magill SS, Edwards JR, Beldavs ZG, Dumyati G, Janelle SJ, Kainer MA, Lynfield R, Nadle J, Neuhauser MM, Ray SM, Richards K, Rodriguez R, Thompson DL, Fridkin SK, Emerging Infections Program Healthcare-Associated Infections and Antimicrobial Use Prevalence Survey Team (2014). Prevalence of antimicrobial use in US acute care hospitals, May–September 2011. JAMA.

[ref-15] Mohamadi J, Havasian MR, Panahi J, Pakzad I (2015). Antifungal drug resistance pattern of *Candida. spp* isolated from vaginitis in Ilam–Iran during 2013–2014. Bioinformation.

[ref-16] Moran C, Grussemeyer CA, Spalding JR, Benjamin Jr DK, Reed SD (2009). *Candida albicans* and non-*albicans* bloodstream infections in adult and pediatric patients: comparison of mortality and costs. The Pediatric Infectious Disease Journal.

[ref-17] Morgan J, Meltzer MI, Plikaytis BD, Sofair AN, Huie-White S, Wilcox S, Harrison LH, Seaberg EC, Hajjeh RA, Teutsch SM (2005). Excess mortality, hospital stay, and cost due to candidemia: a case-control study using data from population-based candidemia surveillance. Infection Control and Hospital Epidemiology.

[ref-18] Nagi M, Tanabe K, Ueno K, Nakayama H, Aoyama T, Chibana H, Yamagoe S, Umeyama T, Oura T, Ohno H, Kajiwara S, Miyazaki Y (2013). The *Candida glabrata* sterol scavenging mechanism, mediated by the ATP-binding cassette transporter Aus1p, is regulated by iron limitation. Molecular Microbiology.

[ref-19] Oberoi JK, Wattal CH, Goel N, Raveendran R, Datta S, Prasad K (2012). Non-albicans Candida species in blood stream infections in a tertiary care hospital at New Delhi, India, Indian. Journal of Medical Research.

[ref-20] Pfaller MA, Jones RN, Messer SA, Edmond MB, Wenzel RP (1998). National surveillance of nosocomial blood stream infection due to *Candida albicans*: frequency of occurrence and antifungal susceptibility in the SCOPE Program. Diagnostic Microbiology and Infectious Disease.

[ref-21] Richardson MD, Warnock DW (1995). Grzybice, rozpoznawanie i leczenie.

[ref-22] Rożkiewicz D (2011). Ręce personelu jako potencjalne źrło zakażń szpitalnych. Zakażenia.

[ref-23] Sanglard D (2016). Emerging threats in antifungal-resistant fungal pathogens. Frontiers in Medicine.

[ref-24] Sanguinetti M, Posteraro B, Lass-Flörl C (2015). Antifungal drug resistance among Candida species: mechanisms and clinical impact. Mycoses.

[ref-25] Swoboda-Kopeć E, Krajewska M, Sulik B, Łuczak M (1998). Oporność klinicznych izolatów *Candida* na leki przeciwgrzybicze. Zakażenia.

[ref-26] Thakur JK, Arthanari H, Yang F, Pan SJ, Fan X, Breger J, Frueh DP, Gulshan K, Li DK, Mylonakis E, Struhl K, Moye-Rowley WS, Cormack BP, Wagner G, Näär AM (2008). A nuclear receptor-like pathway regulating multidrug resistance in fungi. Nature.

[ref-27] Tsai FH, Krol AA, Sarti KE, Bennetti JE (2006). *Candida glabrata* PDR1, a transcriptional regulator of a pleiotropic drug resistance network, mediates azole resistance in clinical isolates and petite mutants. Antimicrobial Agents and Chemotherapy.

[ref-28] Tsimbalari E, Sulik-Tyszka B, Gołaś M, Sikora M, Piskorska K, Swoboda-Kopeć E (2015). Analiza pokrewieństwa genetycznego szczepów *C. glabrata* opornych na leki z grupy azoli wyizolowanych z materiałów klinicznych chorych hospitalizowanych w Samodzielnym Publicznym Centralnym Szpitalu Klinicznym. Postępy Nauk Medycznych.

[ref-29] Vanden Bossche H, Dromer F, Improvisi I, Lozano-Chiu M, Rex JH, Sanglard D (1998). Antifungal drug resistance in pathogenic fungi. Medical Mycology.

[ref-30] Witthuhn F, Toubas D, Beguinot I, Aubert D, Rouger C, Remy G, Pinon JM (1999). Evaluation of the FUNGITEST kit by using strains from human immunodeficiency virus-infected patients: study of azole drug susceptibility. Journal of Clinical Microbiology.

[ref-31] Yildirim M, Sahin I, Kucukbayrak A, Ozdemir D, Tevfik Yavuz M, Oksuz S, Cakir S (2007). Hand carriage of *Candida* species and risk factors in hospital personnel. Mycoses.

[ref-32] Zaidi KU, Mani A, Thawani V, Mehra A (2016). Total protein profile and drug resistance in *Candida albicans* isolated from clinical samples. Molecular Biology International.

