# Supplementary material for: Retrospective observation of drug susceptibility of Candida strains in the years 1999, 2004, and 2015
Source: PeerJ. 2017 Feb 23;5:e3038. doi: 10.7717/peerj.3038 (PMC5326543; doi:10.7717/peerj.3038)
Supplement: File S1 [file peerj-05-3038-s003.docx]

Table 6. Resistance of *Candida albicans* and *non -Candida albicans* strains to one or more antifungal drug

|  | YEAR | | | | P value* |
| --- | --- | --- | --- | --- | --- |
| ***Candida albicans*** | **1999**  **N=282** | **2004**  **N=172** | **2015**  **N= 176** | **Total**  **N=630** | <0.001 |
|  | 101  (35.8%) | 116  (67.4%) | 162  (92%) | 379  (60.2%) |  |
| ***non-Candida albicans*** | **1999**  **N=170** | **2004**  **N=196** | **2015**  **N= 278** | **Total**  **N=644** | <0.001 |
|  | 90  (52.9%) | 126  (64.3%) | 245  (88.1%) | 461  (71.8%) |  |

*Kruskal-Wallis test

Table 7. Drug susceptibility of *Candida albicans* strains to imidazole agents

|  | | Miconazole | Ketoconazole | Itraconazole | Fluconazole | Mean number of strains |
| --- | --- | --- | --- | --- | --- | --- |
| ***Susceptibility/***  ***Year*** | 1999  N=282 | 112  (39.7%) | **114**  **(40.4%)** | 68  (24.1%) | 96  (34%) | 97.5 ± 18.4 |
|  | 2004  N=172 | 12  (16.7%) | 8  (4.6%) | 4  (2.3%) | **29**  **(16.9%)** | 13.25 ± 9.5 |
|  | 2015  N= 176 | **68**  **(38.6%)** | 43  (24.4%) | 17  (11.8%) | 20  (11.4%) | 37 ± 20.5 |
|  | Total  N= 630 | **192**  **(30.5%)** | 165  (26.2%) | 89  (14.1%) | 145  (23%) | 147.75 ± 37.8 |
| P value* | | 0.957 | 0.016 | <0.01 | <0.001 | <0.001 |
|  | | | | | | |
| ***Intermediate/***  ***Year*** | 1999  N=282 | 109  (38.7%) | **110**  **(39%)** | 91  (32.3%) | 36  (12.8%) | 86.5 ± 30.1 |
|  | 2004  N=172 | **94**  **(54.6%)** | 78  (45.3%) | 77  (44.8%) | 71  (41.3%) | 80 ± 8.5 |
|  | 2015  N= 176 | 24  (13.6%) | **39**  **(22.2%)** | 15  (8.5%) | 5  (2.8%) | 22.7 ± 12.5 |
|  | Total  N= 630 | **227**  **(36%)** | **227**  **(36%)** | 183  (29%) | 112  (17.8%) | 187.3 ± 47.01 |
| P value* | | <0.001 | <0.001 | <0.001 | <0.01 | <0.001 |
|  | | | | | | |
| ***Resistance***  ***Year*** | 1999  N=282 | 61  (21.6%) | 58  (20.6%) | 123  (43.6%) | **150**  **(53.2%)** | 98 ± 39.7 |
|  | 2004  N=172 | 66  (38.4%) | 86  (50%) | **91**  **(52.9%)** | 72  (41.9%) | 78.8 ± 10.1 |
|  | 2015  N= 176 | 84  (47.7%) | 94  (53.4%) | 144  (81.8%) | **151**  **(67.1%)** | 118.3 ± 29.6 |
|  | Total  N= 630 | 211  (33.5%) | 238  (37.8%) | 358  (56.8%) | **373**  **(59.2%)** | 295 ± 71.3 |
| P value* | | <0.001 | <0.001 | <0.001 | <0.01 | <0.001 |

*Kruskal-Wallis test

Table 8. Drug susceptibility of *non-Candida albicans* strains to imidazole agents

|  | | Miconazole | Ketoconazole | Itraconazole | Fluconazole | Mean number of strains |
| --- | --- | --- | --- | --- | --- | --- |
| ***Susceptibility/***  ***Year*** | 1999  N=170 | 44  (25.9%) | 22  (12.9%) | 52  (30.6%) | **56**  **(32.9%)** | 43.5 ± 13.1 |
|  | 2004  N=196 | 46  (23.5%) | 52  (25.6%) | 50  (25.5%) | **64**  **(32.7%)** | 53 ± 6.7 |
|  | 2015  N= 278 | **116**  **(56.1%)** | 104  (37.4%) | 109  (39.2%) | 72  (25.9%) | 100.3 ±16.9 |
|  | Total  N= 644 | 206  (32.1%) | 178  (27.7%) | **211**  **(32.9%)** | 192  (29.9%) | 196.8 ± 12.9 |
| P value* | | <0.05 | <0.001 | 0.233 | 0.295 | <0.001 |
|  | | | | | | |
| ***Intermediate/***  ***Year*** | 1999  N=170 | **66**  **(38.8%)** | 24  (14.1%) | 38  (22.4%) | 36  (21.2%) | 41 ± 15.4 |
|  | 2004  N=196 | **96**  **(49%)** | 33  (16.8%) | 32  (16.3%) | 30  (15.3%) | 47.8 ± 27.9 |
|  | 2015  N= 278 | 79  (28.6%) | **84**  **(30.2%)** | 76  (27.3%) | 81  (29.1%) | 80 ±2.9 |
|  | Total  N= 644 | **241**  **(38.6%)** | 141  (22.6%) | 146  (23.4%) | 107  (23.5%) | 158.8 ± 49.8 |
| P value* | | 0.138 | <0.01 | 0.403 | 0.173 | <0.001 |
|  | | | | | | |
| ***Resistance/***  ***Year*** | 1999  N=170 | 60  (35.3%) | **124**  **(72.9%)** | 80  (47.1%) | 78  (45.9%) | **85.5± 23.6** |
|  | 2004  N=196 | 54  (56.3%) | 111  (56.6%) | **114**  **(58.2%)** | 102  (52%) | 95.3 ± 24.2 |
|  | 2015  N= 278 | **83**  **(29.6%)** | 90  (32.4%) | 93  (33.5%) | **125**  **(44.9%)** | 97.8 ± 16.1 |
|  | Total  N= 644 | **197**  **(30.6%)** | **325**  **(50.5%)** | **287**  **(45.9%)** | 227  (36.4%) | **259 ± 50.2** |
| P value* | | 0.399 | <0.05 | 0.07 | 0.999 | <0.001 |

* Kruskal-Wallis test

Table 9. Average number of strains resistant to imidazole by year

|  | YEAR | | | | P value* |
| --- | --- | --- | --- | --- | --- |
| ***Candida albicans*** | **1999**  **N=282** | **2004**  **N=172** | **2015**  **N= 176** | **Total**  **N=630** | <0.001 |
|  | 98 ± 39.7 | 78.8 ± 10.1 | 118.3 ± 29.6 | 295 ± 71.3 |  |
| ***non-Candida albicans*** | **1999**  **N=170** | **2004**  **N=196** | **2015**  **N= 278** | **Total**  **N=644** | <0.001 |
|  | **85.5± 23.6** | 95.3 ± 24.2 | 97.3 ± 16.6 | **259 ± 50.2** |  |

*Kruskal-Wallis test
